# Hole Transport Layer Free Perovskite Light-Emitting Diodes With High-Brightness and Air-Stability Based on Solution-Processed CsPbBr_3_-Cs_4_PbBr_6_ Composites Films

**DOI:** 10.3389/fchem.2022.828322

**Published:** 2022-01-21

**Authors:** Fang Yuan, Min Zhang, Chunrong Zhu, Xiaoyun Liu, Chenjing Zhao, Jinfei Dai, Hua Dong, Bo Jiao, Xuguang Lan, Zhaoxin Wu

**Affiliations:** ^1^ Key Laboratory for Physical Electronics and Devices of the Ministry of Education and Shaanxi Key Lab of Information Photonic Technique, School of Electronic and Information Engineering, Xi’an Jiaotong University, Xi’an, China; ^2^ Institute of Artificial Intelligence and Robotics, Xi’an Jiaotong University, Xi’an, China; ^3^ Collaborative Innovation Center of Extreme Optics, Shanxi University, Taiyuan, China

**Keywords:** all-inorganic perovskites, perovskite light-emitting diodes, dual-phase, CsPbBr_3_-Cs_4_PbBr_6_ composites, device stability

## Abstract

Recently, perovskite light-emitting diodes (PeLEDs) have drew widespread attention due to their high efficiencies. However, because of the sensitivity to moisture and oxygen, perovskite luminescent layers are usually prepared in high-purity nitrogen environment, which increases the cost and process complexity of device preparation and seriously hindrances its commercialization of PeLED in lighting and display application. Herein, dual-phase all-inorganic composite CsPbBr_3_-Cs_4_PbBr_6_ films are fabricated from CsBr-rich perovskite solutions by a simple one-step spin-coating method in the air with high humidity. Compared with the pure CsPbBr_3_ film, the composite CsPbBr_3_-Cs_4_PbBr_6_ film has much stronger photoluminescence emission and longer fluorescence lifetime, accompanied by increased photoluminescence quantum yield (33%). As a result, we obtained green PeLED devices without hole transport layer exhibiting a maximum brightness of 72,082 cd/m^2^ and a maximum external quantum efficiency of about 2.45%, respectively. More importantly, the champion device shows excellent stability with operational half-lifetime exceeding 1,000 min under continuous operation in the air. The dual-phase all-inorganic composite CsPbBr_3_-Cs_4_PbBr_6_ film shows attractive prospect for advanced light emission applications.

## Introduction

As a star material, to date, metal halide perovskites have attracted considerable interests in optoelectronic applications owing to their facile solution-processed ability (Li et al., 2017; Liu et al., 2020), tunable band-gap (Van Le et al., 2018), high carrier mobility (Shen et al., 2020), and high photoluminescence quantum yield (PLQY) (Lu et al., 2019). Since the first realization of perovskite light-emitting diodes (PeLEDs) at room temperature pioneered by R. Friend and his coworkers in 2014 (Tan et al., 2014), the efficiency of PeLEDs has improved dramatically in just a few years, with the external quantum efficiencies (EQEs) all exceeding 20% in green, red and near-infrared region (Zhang L. et al., 2017; Cao et al., 2018; Lin et al., 2018; Zhao et al., 2018; Fang et al., 2020). However, subject to moisture and oxygen sensitivity (Cho et al., 2018), high-quality perovskite films often need to be prepared in high-purity nitrogen environment, which increases the production cost and process complexity of PeLED devices and seriously restricts their commercial applications in lighting and display. In addition, the instability of perovskite materials and their PeLED devices also limits their development and application (Cho et al., 2018). Therefore, it is very desirable to directly prepare efficient and stable perovskite films in atmospheric environment.

So far, compared to the organic-inorganic lead halide perovskite materials with a three-dimensional APbX_3_ structure (where A for CH_3_NH_3_^+^ or HC(NH_2_)_2_^+^ cation; X for I^−^, Br^−^, Cl^−^ halide ion), which prone to degradation under moisture, oxygen, heat, and illumination exposure (Cho et al., 2018), all-inorganic perovskites such as CsPbX_3_ have drew widespread attention due to their excellent stability and high PLQY (Shen et al., 2020; Yang et al., 2020; Lin et al., 2021). However, mainly derived from the low solubility of CsBr precursor in commonly used solvents such as dimethyl sulfoxide (Zhang et al., 2016), all-inorganic perovskite films prepared by one-step solution-processed method are often rough and discontinuous with pinholes, which leads to large leakage current, thus compromising device efficiency and stability. For high-performance PeLEDs, many efforts have been devoted to optimizing solution-processed perovskite films such as organic ligands modification (Wang et al., 2016; Xiao et al., 2017; Yang et al., 2018), alkali-metal ions doping (Abdi-Jalebi et al., 2018; Zhang et al., 2020), structure-dimensionality reduction (Xing et al., 2018; Zhu et al., 2021), stoichiometry control (Cho et al., 2017), construction of nanocrystals (Wang et al., 2018) and so on. Moreover, electric-field-induced ion migration has been demonstrated to significantly restrict the stability of PeLED devices, which requires a new structural design strategy (Zhang et al., 2020; Woo et al., 2021). Obviously, it is very important to explore the preparation of high-quality all-inorganic perovskite films in air towards efficient and stable PeLED devices to promote its commercialization process.

In this work, by regulating the molar ratios of PbBr_2_ and CsBr, the dual-phase all-inorganic composite CsPbBr_3_-Cs_4_PbBr_6_ films without pinholes are fabricated from CsBr-rich solutions by a simple one-step spin-coating method in the air with high humidity. Compared with the pure CsPbBr_3_ film, the composite CsPbBr_3_-Cs_4_PbBr_6_ film has much longer lifetime with increased PLQY of about 33%. Based on the optimized composite CsPbBr_3_-Cs_4_PbBr_6_ films, combined with LiF interlayer modification, the champion PeLED device without hole transport layer exhibits a maximum brightness of 72,082 cd/m^2^ with a current density (CE) of ≈7.67 cd/A. More interestingly, the champion device exhibits robust durability with the half-lifetime over 1,000 min under continuous operation in the air at an initial luminance of about 100 cd/m^2^. The dual-phase all-inorganic composite CsPbBr_3_-Cs_4_PbBr_6_ film and LiF interlayer modification provides a useful idea for designing PeLED devices with high air-stability.

## Materials and Methods

### Materials

Lead bromide (PbBr_2_, 99.999%, Sigma Aldrich), Cesium bromide (CsBr, 99.999%, Alfa Aesar), Dimethyl sulfoxide (DMSO, 99.8 wt%, Alfa Aesar), PEDOT: PSS (Clevios Al4083, Haraeus), Bathophenanthroline (Bphen, 99%, Nichem), Lithium fluoride (LiF, 99.99%, Nichem) were used as received without further purification.

### Preparation of Perovskite Films

The perovskite precursor solutions were prepared by dissolving 0.35 M PbBr_2_ and the corresponding amount of CsBr in DMSO with different PbBr_2_: CsBr molar ratios = 1:0.8, 1:1.0, 1:1.1, 1:1.3, 1:1.5, and 1:1.7, respectively. The precursor solutions were stirred in a high-purity nitrogen-filled glove box at room temperature for 4 h. And then the solutions were stand for 1 h at room temperature, precipitates were formed in the CsBr-rich solutions (PbBr_2_: CsBr = 1:1.3, 1:1.5, 1:1.7), top transparent solutions were decanted for using. The perovskite films were all prepared by a facile one-step spin-coating method without anti-solvent assist. Before preparing the perovskite films, the glass substrates were cut into a suitable size, cleaned with deionized water and organic solvents, dried under a hot lamp, and treated in an ultraviolet ozone environment for 10 min. Subsequently, the perovskite precursor solutions were spin-coated at 1,000 rpm for 60 s in the air with high humidity (∼60%). Finally, the samples were annealed at 200°C for 60 s to obtain perovskite films in the atmosphere.

### Devices Fabrication

The structure of the PeLED devices is ITO/Perovskite/LiF (8 nm)/Bphen (60 nm)/LiF (1 nm)/Al (100 nm). Emission area of the devices was about 12 mm^2^. To prepare the devices, the indium-tin oxide (ITO)-coated patterned glass substrates were cleaned with deionized water and organic solvents, and then exposed to a UV–ozone environment for 10 min. For the preparation of the perovskite films, the aforementioned precursor solutions (about 0.25 ml) were spin-coated at 1,000 r.p.m for 60 s onto the clean ITO-coated substrates and then annealed at 200°C for 60 s in the air (humidity ∼60%) to form the perovskite films. Finally, the aforementioned substrates with perovskite films were transported into a thermal evaporation chamber for the deposition of 8 nm LiF, 60 nm Bphen, 1 nm LiF, and 100 nm Al, sequentially. The evaporation rates for LiF, Bphen, and Al were 0.2, 1.5, and 4 Å/s, respectively. The basic pressure of vapor deposition is 1 × 10^−3^ Pa. Film thickness was determined *in-situ* by a quartz-crystal sensor and confirmed by a profilometer.

### Characterization

The surface of the all-inorganic perovskite films was investigated by scanning electron microscopy (SEM, Quanta 250, FEI). The surface morphology was measured by atomic force microscope (AFM, NT-MDT, Russia). The crystalline structure was measured by X-ray diffraction (XRD, D/MAX-2400, Japan, Rigaku) with Cu-Kα radiation. The absorption spectra and the PL spectra were obtained by an ultraviolet-visible spectrophotometer (HITACHI U-3010, Japan) and a fluorescence spectrophotometer (Fluoromax-4 spectrofluometer), respectively. Time-resolved PL spectra were recorded with a 100 ps time resolution using a time-correlated single photon counting (TCSPC) system (FLS920 spectrometer) (excited by picosecond pulsed LEDs, pulse duration: <850 ps, repetition rate: 10 MHz). Photoluminescence quantum yield was tested using FLS920 spectrometer with an integrating sphere. A cross-sectional SEM image of the champion PeLED device is obtained by scanning electron microscopy (SEM, Quanta 250, FEI). The luminance-current-voltage (*L*-*I*-*V*) characteristics of blue PeLEDs were measured using a computer-controlled source meter (Keithley 2602) and a calibrated silicon photodiode (integrated over 1 s). The electroluminescence spectra were measured by PR650 spectrometer. For the stability of the champion PeLED device, the half-lifetime (50% decay) was measured at an initial luminance of about 100 cd/m^2^ under continuous constant current supplied by Keithley 2602. All measurements were carried out at room temperature in atmospheric environment. It should be noted that, the luminance (cd/m^2^) was directly measured using a computer-controlled source meter (Keithley 2602) and a calibrated silicon photodiode (integrated over 1 s). The current density (mA/cm^2^) was calculated by dividing the current (mA) flowing in the device by the effective area (about 12 mm^2^). The current efficiency (cd/A) was calculated by dividing (the luminance (cd/m^2^) × the effective area) by the current I (A). The EQE is the ratio between the number of photons emitted from the device and the electrons injected into the device. Thus, the EQE will be:

EQE= ∑λ(nm)ΦhcλI×e

where, *Φ* is the total spectral power, *h* is the Planck constant, *c* is the speed of light, *λ* is the electroluminescent wavelength and *e* is the unit charge.

## Results and Discussion

**FIGURE 1:**
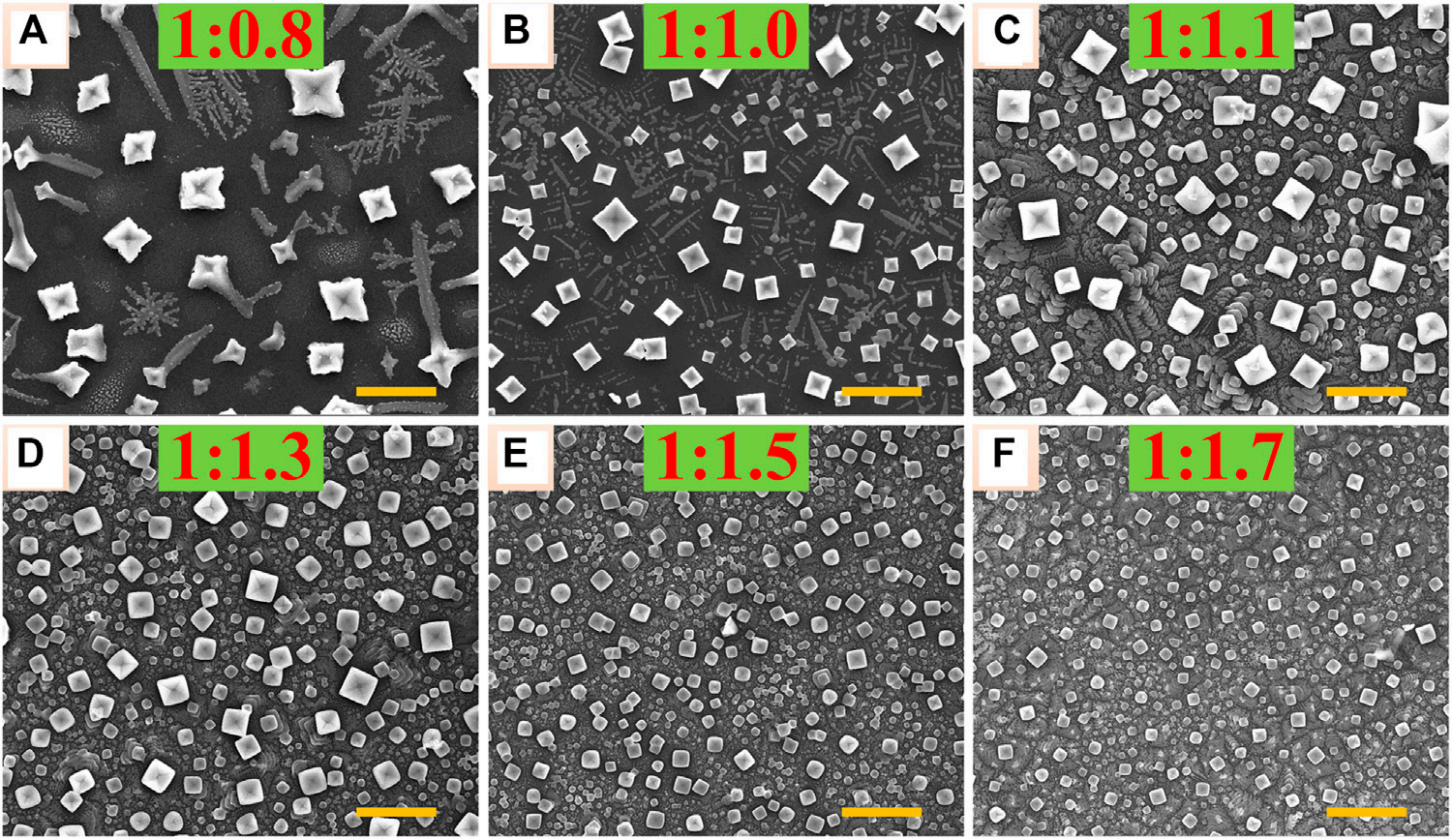
Top view SEM images of the corresponding all-inorganic perovskite films with different molar ratios of PbBr_2_ and CsBr, such as **(A)** 1:0.8, **(B)** 1:1.0, **(C)** 1:1.1 **(D)** 1:1.3, **(E)** 1:1.5, **(F)** 1:1.7, respectively. The scale bar is 2 μm in all images.

The all-inorganic perovskite films were all prepared by a facile one-step spin-coating method in the air with high humidity using the perovskite precursor solutions dissolving PbBr_2_ and CsBr with different molar ratios = 1:0.8, 1:1.0, 1:1.1, 1:1.3, 1:1.5, and 1:1.7, respectively, as explained in the MATERIALS AND METHODS. In order to study the influence of different molar ratios of PbBr_2_ and CsBr on the morphology of all-inorganic perovskite films, the scanning electron microscopy (SEM) analysis was carried out. As shown in Figures 1A–F, with the increase of CsBr ratios, the grain size of perovskite decreases and the film coverage rate increases gradually. Specifically, as shown in Figure 1A, when the molar ratio of PbBr_2_ to CsBr is 1:0.8, the crystallinity of perovskite crystals is very poor with dendritic grains. As the molar ratio of CsBr increases, the grain size decreases significantly. It can be inferred that the decreasing trend of the perovskite grain size is basically consistent with the increase of CsBr ratios. Especially, when the CsBr is excessive, as shown in Figures 1D–F, the film coverage rate increases obviously with perovskite grain size decreasing to about 50–400 nm. The 3D AFM image of perovskite film with the molar ratio of PbBr_2_ to CsBr = 1:1.5 shows flat and compact morphology with the root-mean-square (RMS) roughness of about 24 nm (Supplementary Figure S1). It should be noted that, due to the poor solubility of CsBr, when the molar ratio of PbBr_2_ to CsBr exceeds 1:1.3, the perovskite precursor solutions cannot be completely dissolved.

**FIGURE 2:**
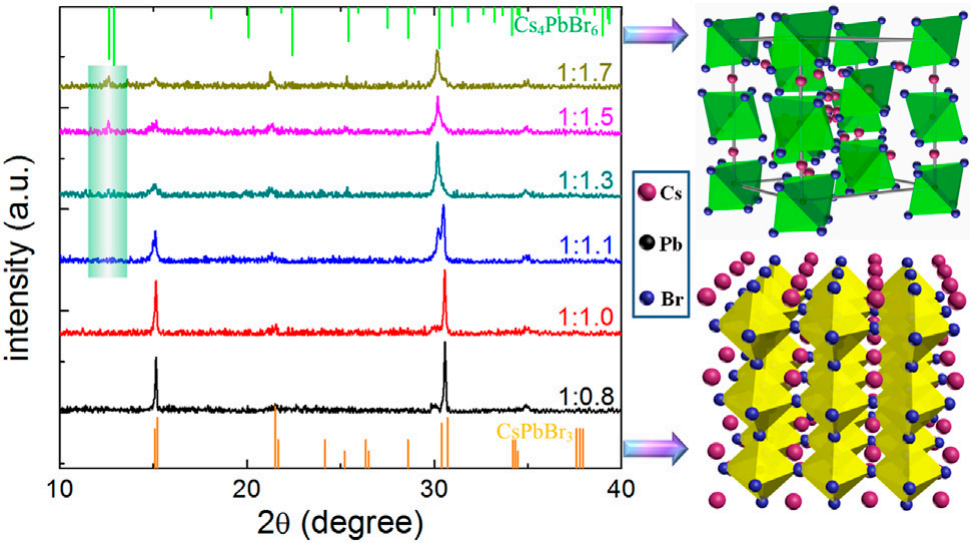
Bragg-Brentano X-ray diffraction patterns for the corresponding all-inorganic perovskite films with different molar ratios of PbBr_2_ and CsBr, such as 1:0.8, 1:1.0, 1:1.1, 1:1.3, 1:1.5, 1:1.7, respectively. The right of the figure shows the crystal structures of CsPbBr_3_ (bottom) and Cs_4_PbBr_6_ (top) perovskite.

In order to analyze the crystallization properties of perovskite films with different molar ratios of PbBr_2_ and CsBr, XRD measurements were further carried out. As shown in Figure 2, all the XRD patterns show diffraction peaks at 15.09°, 21.36°, and 30.52°, which can be assigned to (100), (101), and (200) facets of the typical three-dimensional CsPbBr_3_ perovskite structure in the cubic phase, respectively (Akkerman et al., 2017; Nasi et al., 2020). Obviously, with the increase of CsBr ratios, the XRD peaks corresponding to CsPbBr_3_ weaken, indicating smaller grain size in the films, which is consistent with the SEM results (Figure 1). In particular, when the molar ratio of PbBr_2_ to CsBr exceeds 1:1.3, new XRD peaks appear at 12.54° and 25.41°, corresponding to (110) and (220) facets of the typical zero-dimensional Cs_4_PbBr_6_ perovskite structure in the rhombohedral phase (Zhang Y. et al., 2017). It can be seen that, as for the CsBr-rich perovskite precursor solutions, dual-phase all-inorganic composite CsPbBr_3_-Cs_4_PbBr_6_ films were eventually formed. The energy dispersion spectroscopy (EDS) result of the all-inorganic perovskite film with the molar ratio of PbBr_2_ to CsBr = 1:1.5 shows the elemental ratio of Cs/Pb/Br is about 1.42:1:3.59 (Supplementary Figure S2), which also indicates the formation of a dual-phase all-inorganic composite CsPbBr_3_-Cs_4_PbBr_6_. The excessive CsBr on the surface of the perovskite films reacts with partial CsPbBr_3_ to form Cs_4_PbBr_6_, which is agreement with the reported literature (Saidaminov et al., 2016; Akkerman et al., 2017).

**FIGURE 3:**
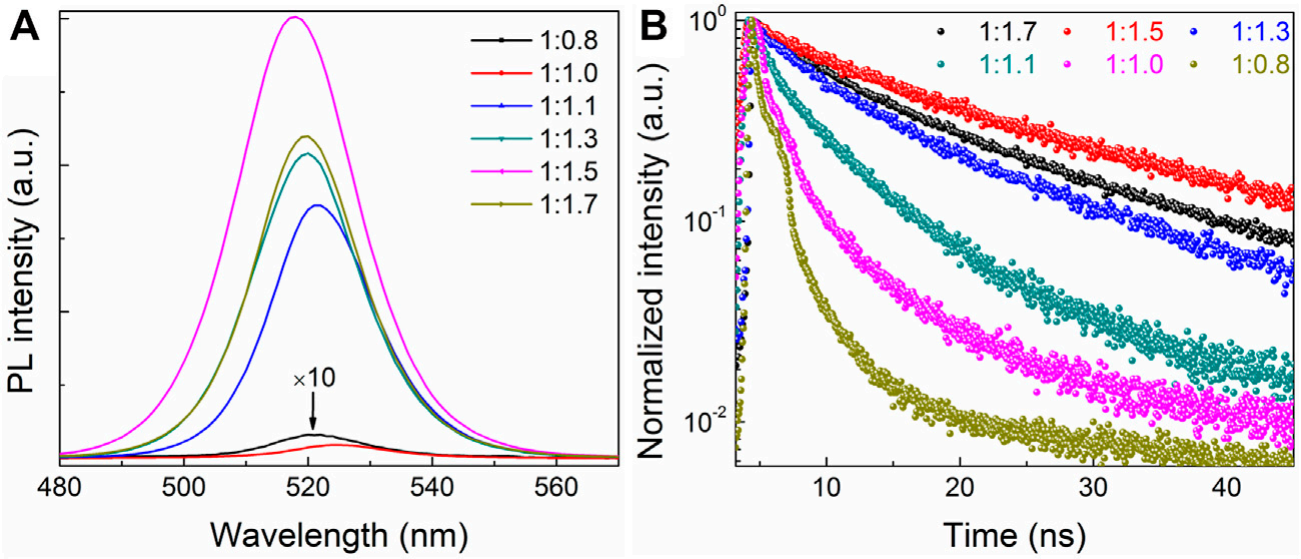
**(A)** The steady-state PL spectra and **(B)** time-resolved photoluminescence (TRPL) measurements of the corresponding all-inorganic perovskite films with different molar ratios of PbBr_2_ and CsBr, such as 1:0.8, 1:1.0, 1:1.1, 1:1.3, 1:1.5, 1:1.7, respectively.

To investigate the effects of different CsBr ratios on the optical properties of all-inorganic perovskite films, the PL spectra of perovskite films with different molar ratios of PbBr_2_ and CsBr were measured, as shown in Figure 3A. It can be seen that, as the proportion of CsBr increases, the PL intensity of the perovskite films increases. When the molar ratio of PbBr_2_ to CsBr is 1:1.5, the PL intensity is the highest with the PLQY of about 33%. In other words, compared with the pure CsPbBr_3_ film, the dual-phase composite CsPbBr_3_-Cs_4_PbBr_6_ film has better PL characteristics. When the molar ratio of PbBr_2_ to CsBr exceeds 1:1.5, the PL intensity of perovskite films decreases slightly. In addition, it can be seen intuitively that, with the increase of CsBr ratios, the PL emission peaks continuously blue shift passably derived from the change of crystal structure and film morphology, which is consistent with XRD and SEM results. The alignment of [PbBr_6_]^4−^ octahedron sublattices in the composite CsPbBr_3_-Cs_4_PbBr_6_ film dictates the optical transition behavior (Saparov and Mitzi, 2016). In order to further study the effect of different CsBr ratios on the fluorescence lifetime of all-inorganic perovskite films, the time-resolved photoluminescence (TRPL) measurements were carried out, as shown in Figure 3B, which shows that all the prepared samples have a double exponential decay behavior. The TRPL decay curves are fitted by the double decay function, and the average lifetime is obtained from the double exponential decay function, as summarized in Supplementary Table S1. Obviously, as the ratio of CsBr gradually increases, the average lifetime *τ*_ave_ of perovskite films first increases from 1.93 to 18.88 ns, and then decreases to 16.64 ns when the CsBr proportion exceeding 1.7, which is consistent with PL analysis as shown in Figure 3A. These results suggest that excessive CsBr can effectively passivate the defects of perovskite, which will effectively inhibit the non-radiative recombination channels, and then enhance the PLQY of composite CsPbBr_3_-Cs_4_PbBr_6_ films. It is proved that, compared with the pure CsPbBr_3_ film, the dual-phase composite CsPbBr_3_-Cs_4_PbBr_6_ film has much higher PLQY and longer fluorescence lifetime, which is beneficial to the improvement of electroluminescent device efficiency.

**FIGURE 4:**
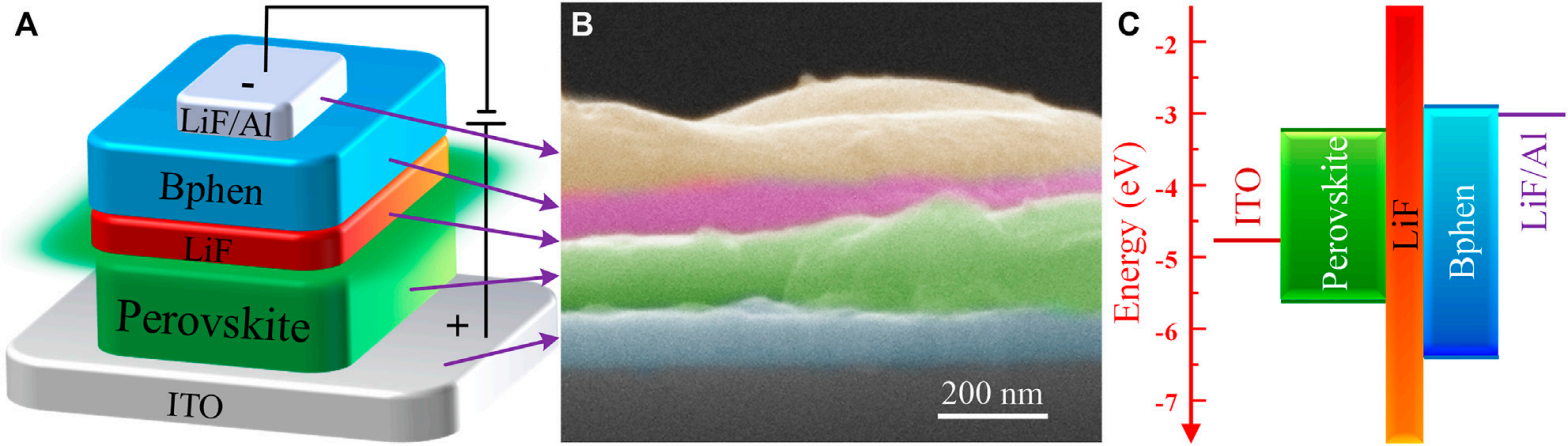
**(A)** Device configuration and **(B)** cross sectional SEM image of the PeLED based on the all-inorganic perovskite film with the molar ratio of PbBr_2_ to CsBr = 1:1.5. **(C)** Energy band diagram of the perovskites and PeLED devices.

Based on the above-mentioned all-inorganic perovskite films with different molar ratios of PbBr_2_ and CsBr, the green PeLEDs are then fabricated. Inspired by the “Insulator-Perovskite-Insulator” structure reported in details elsewhere in our previous work which could effectively induce charge carriers into perovskite crystals and block leakage currents *via* pinholes simultaneously (Shi et al., 2018; Yuan et al., 2018; Yuan et al., 2020; Zhang et al., 2020), the device structure used here is ITO/Perovskite/LiF (8 nm)/Bathophenanthroline (Bphen, 60 nm)/LiF (1 nm)/Al (100 nm), and the schematic diagram of device configuration is shown in Figure 4A. The cross-sectional SEM image of PeLED based on the all-inorganic perovskite film with the molar ratio of PbBr_2_ to CsBr = 1:1.5 is shown in Figure 4B. Each functional layer of the PeLED device can be cleanly observed with a flat contact surface. The energy level diagram of the perovskites and PeLED devices is shown in Figure 4C. It should be pointed out that, we prepared the luminescent perovskite layers directly on ITO substrates without hole transport layer here. As a material with bipolar injection characteristics, the all-inorganic perovskite layer acts as both hole transport layer and emitting layer, and the holes and electrons radiatively recombine between the perovskite layer and the electron transport layer (Bphen). For the thin LiF layer embedded between perovskite and Bphen, its main role is to accumulate holes in perovskite layer, effectively avoid luminescence quenching caused by the Bphen layer and greatly suppress leakage current, thus improve the device performance (Shi et al., 2018).

**FIGURE 5:**
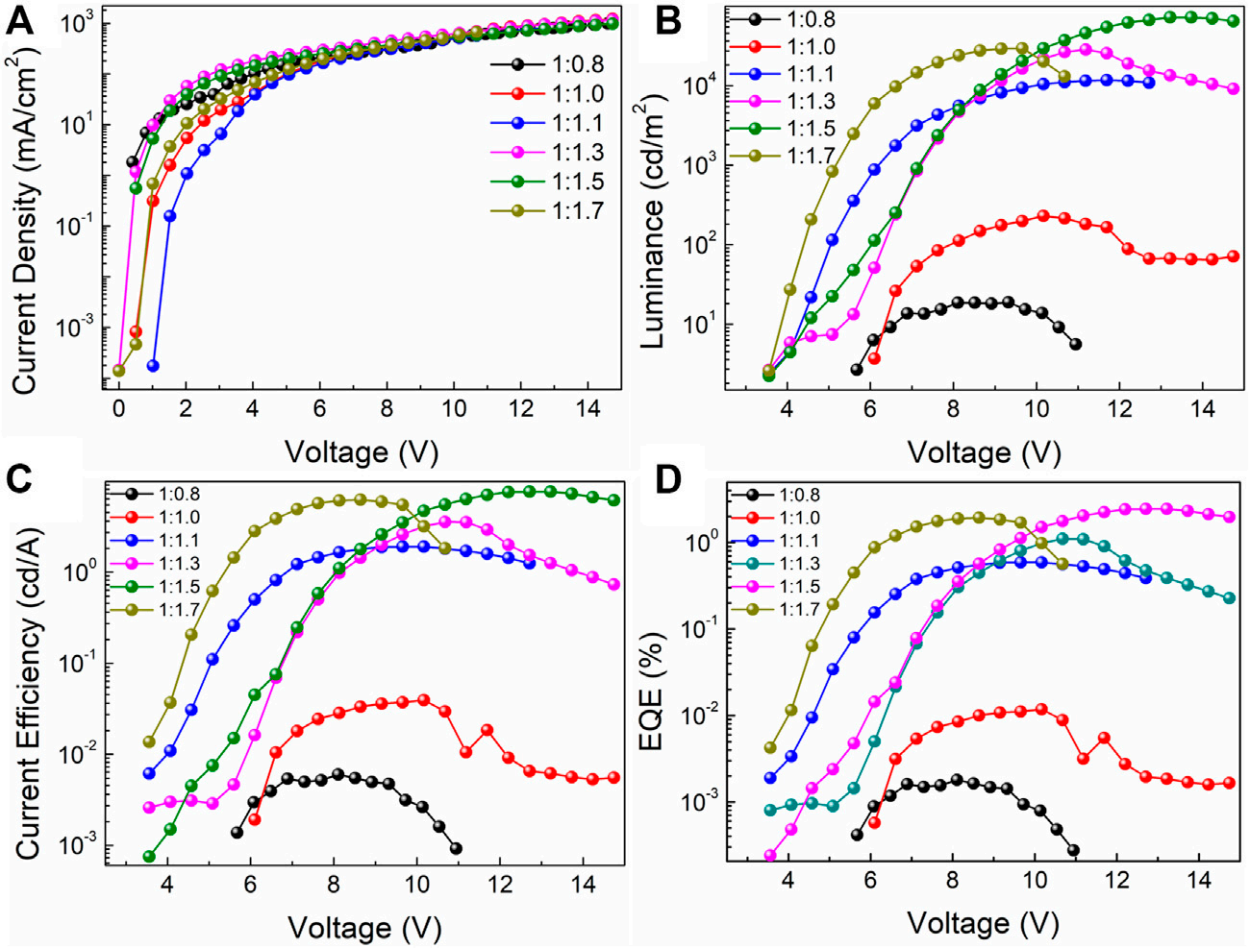
**(A)** Current density-voltage (*J-V*), **(B)** luminance-voltage (*L-V*), **(C)** current efficiency-voltage (*CE-V*), **(D)** EQE-voltage (*EQE-V*) of the PeLED devices based on the perovskite films doped with different molar ratios of PbBr_2_ and CsBr, such as 1:0.8, 1:1.0, 1:1.1, 1:1.3, 1:1.5, 1:1.7, respectively.

The electroluminescent characteristic curves of the PeLEDs based on the all-inorganic perovskite films with different molar ratios of PbBr_2_ and CsBr are shown in Figures 5A–D. Table 1 collects the performance parameters of all the PeLED devices based on the corresponding perovskite films with the structure of ITO/Perovskite/LiF/Bphen/LiF/Al. From the current density-voltage (*J*-*V*) characteristic curves shown in Figure 5A, the current density is relatively high when the molar ratio of PbBr_2_ to CsBr is 1:1.5, suggesting efficient carrier injection. Besides, as the luminance-voltage (*L*-*V*) curve shown in Figure 5B, the turn-on voltage decreases from ∼5.8 V for the device with the molar ratio of PbBr_2_ to CsBr = 1:0.8–∼3.5 V for the CsBr-rich devices, which also indicates more efficient carrier injection. With the increase of CsBr ratios, the brightness of the corresponding device first increases and then decreases when the CsBr percentage exceeding 1.5 (Table 1), which is consistent with the PL characteristics of the perovskite films. The current efficiency-voltage (*CE*-*V*) and EQE-voltage (EQE-*V*) characteristic curves of the PeLED devices are shown in Figures 5C,D. Apparently, the maximum current efficiency and EQE are significantly enhanced with the dual-phase all-inorganic composite CsPbBr_3_-Cs_4_PbBr_6_ films. Particularly, the device with the molar ratio of PbBr_2_ to CsBr = 1:0.8 shows the maximum luminance, CE and EQE of 19 cd/m^2^, 0.006 cd/A, and 0.001%, respectively. While the champion device based on the all-inorganic perovskite film with the molar ratio of PbBr_2_ to CsBr = 1:1.5 exhibits significantly improved luminance, CE and EQE of 72,082 cd/m^2^, 7.67 cd/A, and 2.45%, respectively (Table 1). The significantly improved device performance can be mainly attributed to the improved luminescent characteristics of the dual-phase composite CsPbBr_3_-Cs_4_PbBr_6_ films generated by excessive CsBr, such as high PLQY, low defect density and compact film morphology, effectively inhibiting non-radiative recombination and reducing leakage current, enhancing carrier injection, thereby improving the luminous efficiency of the PeLED devices. More importantly, the introduction of 8 nm LiF layer is critical to the improvement of device performance. For devices without 8 nm LiF layer, that is, with the structure of ITO/Perovskite/Bphen (60 nm)/LiF (1 nm)/Al (100 nm), based on the optimized all-inorganic perovskite film, the optimized PeLED shows drastically reduced performance, exhibiting a maximum luminance, CE and EQE of 45,708 cd/m^2^, 3.63 cd/A, and 1.16%, respectively (Supplementary Figure S3).

**TABLE 1:** Device performance parameters of the prepared PeLED devices based on the corresponding perovskite films with different molar ratios of PbBr_2_ and CsBr, such as 1:0.8, 1:1.0, 1:1.1, 1:1.3, 1:1.5, 1:1.7, respectively.

Devices PbBr_2_:CsBr	EL peak (nm)	Max. L (cd/cm^2^)	Max. CE (cd/A)	Max. EQE (%)
1:0.8	523	19	0.006	0.001
1:1.0	525	230	0.04	0.012
1:1.1	522	11,725	1.91	0.59
1:1.3	520	28,324	3.57	1.11
1:1.5	518	72,082	7.67	2.45
1:1.7	519	29,358	6.27	1.94

**FIGURE 6:**
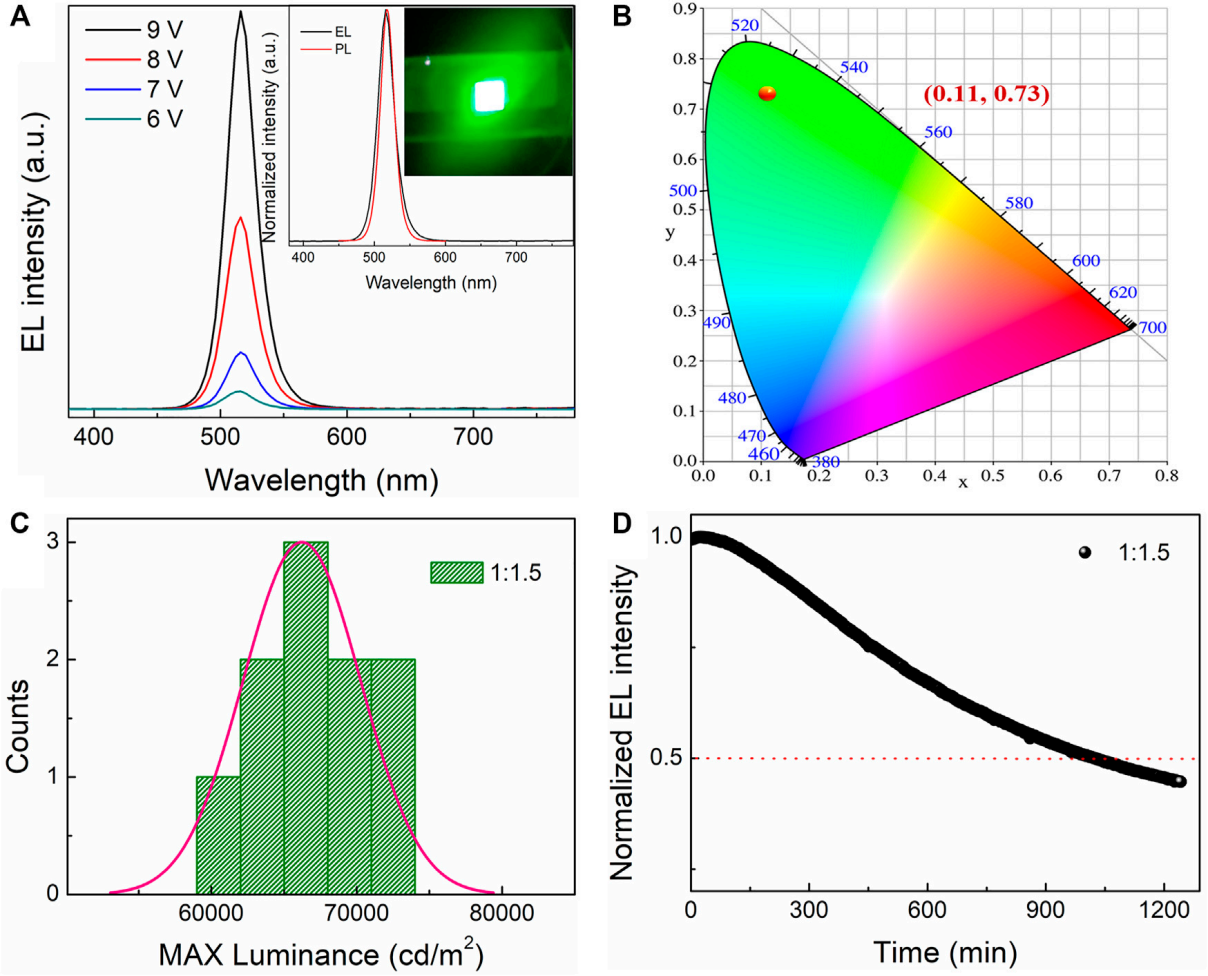
**(A)** EL spectra of the PeLED device based on the all-inorganic perovskite film with the molar ratio of PbBr_2_ to CsBr = 1:1.5 operating under different applied voltages. The inset shows its normalized EL and PL spectra, as well as its digital photographs in operation at 6 V. **(B)** CIE chromaticity coordinate diagram, **(C)** histogram of maximum luminance, **(D)** stability test under continuously operational conditions for the PeLED device based on the all-inorganic CsPbBr_3_-Cs_4_PbBr_6_ perovskite film with the molar ratio of PbBr_2_ to CsBr = 1:1.5.

In addition to the high EL performance, the PeLED device based on the dual-phase all-inorganic composite CsPbBr_3_-Cs_4_PbBr_6_ with the molar ratio of PbBr_2_ to CsBr = 1:1.5 shows high operational stability and high reproducibility. Figure 6A shows the EL spectra of the PeLED device based on the all-inorganic perovskite film with the molar ratio of PbBr_2_ to CsBr = 1:1.5 collected under different applied voltages, showing constant EL peak positions at 518 nm. It shows bright and homogeneous green emission throughout the whole active device area from its representative photograph of operating PeLED device at 6 V, as shown in the inset in Figure 6A, and its EL spectrum almost coincides with its PL spectrum, demonstrating the outstanding spectral stability. Figure 6B shows the CIE chromaticity coordinate diagram of the PeLED device based on the all-inorganic perovskite film with the molar ratio of PbBr_2_ to CsBr = 1:1.5, enabling a CIE 1931 chromatic coordinate as (0.11, 0.73). These PeLED devices also show a high reproducibility (Figure 6C), which originates from the superior film-forming ability for the CsBr-rich solutions. High operational stability is the base of long-term stability of PeLEDs, which is critical for lighting and displaying applications. As shown in Figure 6D, the operational stability of the champion device was further measured by testing the durable time of 50% loss in EL intensity under continuously working conditions. Herein, the device lifetime measurement was operated with the initial luminance of about 100 cd/m^2^, and it was observed that the device shows a rapid 50% loss of its initial EL intensity after 1,030 min. It is worthy pointed out that, compared to the PeLED devices with traditional hole transport layers such as poly (3,4-ethylenedioxythiophene) polystyrene sulfonate (PEDOT:PSS), the structure without hole transport layer of ITO/Perovskite/LiF/Bphen/LiF/Al is designed here on purpose to effectively improve the operational stability. Due to the huge barriers induced by the compact inorganic interlayer and insulating layers on both sides of perovskite layer, the structure used here could significantly suppress the electric-field-induced ion migrations of the perovskites, thus greatly improving the device stability (Zhang et al., 2020).

## Conclusion

In conclusion, dual-phase all-inorganic composite CsPbBr_3_-Cs_4_PbBr_6_ films were prepared from CsBr-rich solutions by a simple one-step spin-coating method in the air with high humidity, which possess higher PLQY, longer fluorescence lifetime, and dense film morphology compared to the pure CsPbBr_3_ films. Combined with LiF interlayer modification, the optimized PeLED based on the composite CsPbBr_3_-Cs_4_PbBr_6_ film with the molar ratio of PbBr_2_ to CsBr = 1:1.5 has a brightness of 72,082 cd/cm^2^ at 518 nm, a maximum current efficiency of 7.67 cd/A, and a maximum EQE of 2.45%, respectively. In addition, the half-decay lifetime of champion device exceeds 1,000 min under continuous operation in the air. This work suggests a feasible way to improve the performance of PeLEDs in the air, paving the way for the practical application of PeLEDs.

## Data Availability

The original contributions presented in the study are included in the article/Supplementary Material, further inquiries can be directed to the corresponding author.
