# Supplementary material for: Hole Transport Layer Free Perovskite Light-Emitting Diodes With High-Brightness and Air-Stability Based on Solution-Processed CsPbBr3-Cs4PbBr6 Composites Films
Source: Front Chem. 2022 Jan 21;10:828322. doi: 10.3389/fchem.2022.828322 (PMC8814343; doi:10.3389/fchem.2022.828322)
Supplement: Supplementary file 1 [file DataSheet1.docx]

**Supplementary Material**


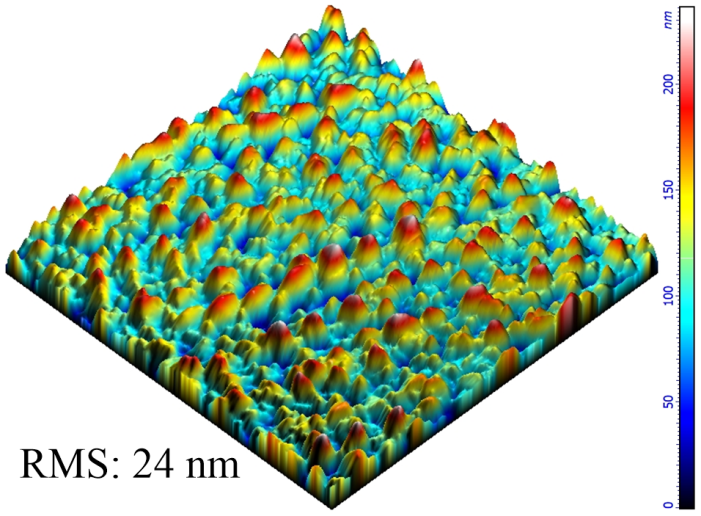


**Figure S1.** The 3D AFM image of the all-inorganic perovskite film with the molar ratio of PbBr_2_ to CsBr = 1:1.5 over the measured area of 10 μm × 10 μm.


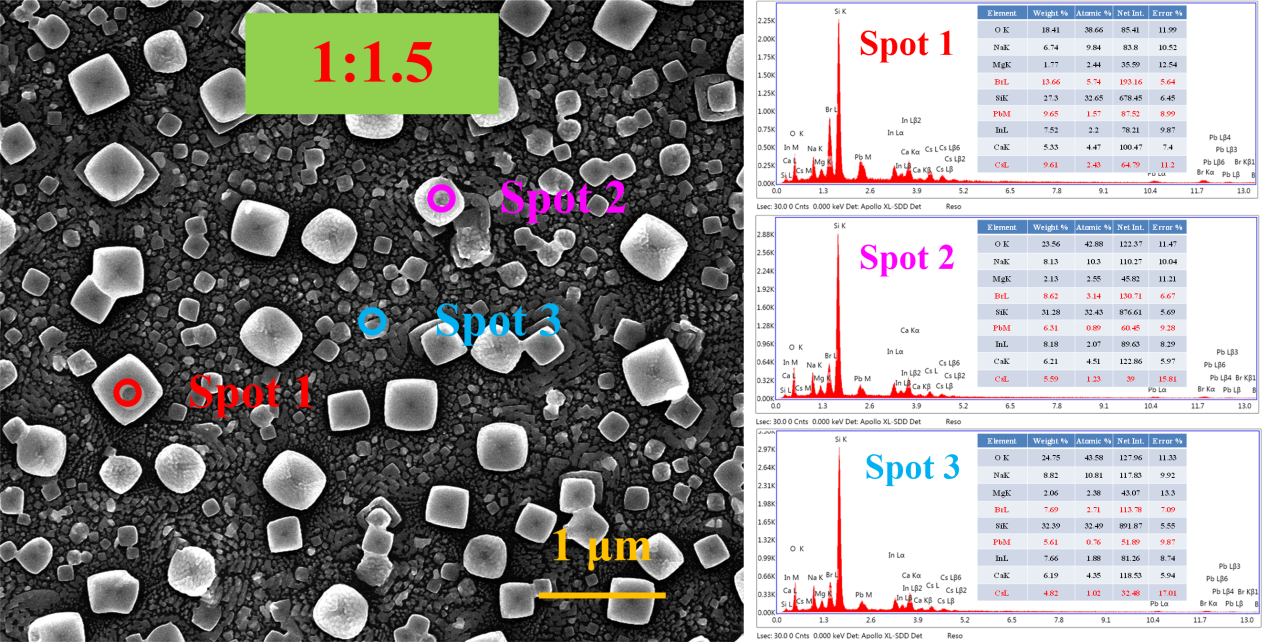


**Figure S2.** The energy dispersion spectroscopy (EDS) spectra of three different spots from the all-inorganic perovskite film with the molar ratio of PbBr_2_ to CsBr = 1:1.5, showing average elemental ratio of Cs/Pb/Br is about 1.42:1:3.59.

**
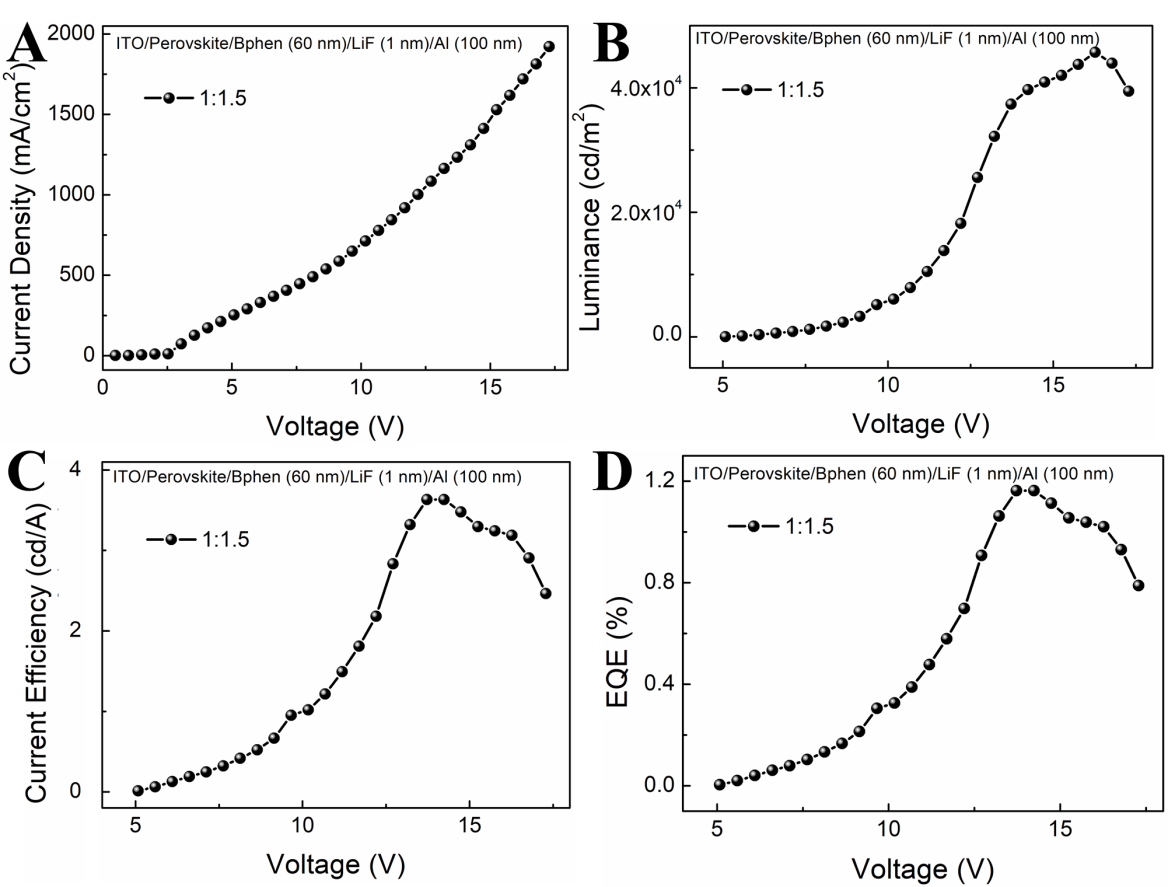
**

**Figure S3.** **(A)** Current density-voltage (*J-V*), (e) luminance-voltage (*L-V*), (f) current efficiency-voltage (*CE-V*), (g) EQE-voltage (*EQE-V*) of the optimized PeLED devices with the structure of ITO/Perovskite/Bphen (60 nm)/LiF (1 nm)/Al (100 nm), based on the all-inorganic perovskite film with the molar ratio of PbBr_2_ to CsBr = 1:1.5.

**Table S1.** PL lifetime obtained from fitted time-resolved PL decay data of the all-inorganic perovskite films with different molar ratios of PbBr_2_ and CsBr.

| **PbBr_2_:CsBr** | **τ_1_ (ns)** | **f_1_ (%)** | **τ_2_ (ns)** | **f_2_ (%)** | **χ^2^** | **τ_ave_ (ns)** |
| --- | --- | --- | --- | --- | --- | --- |
| **1:0.8** | **0.51** | **35.9** | **2.73** | **64.1** | **1.092** | **1.93** |
| **1:1.0** | **0.79** | **25.84** | **5.58** | **74.16** | **1.079** | **4.34** |
| **1:1.1** | **2.02** | **23.72** | **7.89** | **76.28** | **1.072** | **6.5** |
| **1:1.3** | **4.45** | **21.12** | **15.64** | **78.88** | **1.048** | **13.28** |
| **1:1.5** | **5.32** | **9.25** | **20.26** | **90.75** | **1.017** | **18.88** |
| **1:1.7** | **5.14** | **18.63** | **19.28** | **81.37** | **1.015** | **16.64** |
